# Detection *of adeABC* efllux pump encoding genes and antimicrobial effect of *Mentha longifolia* and *Menthol* on MICs of imipenem and ciprofloxacin in clinical isolates of *Acinetobacter baumannii*

**DOI:** 10.1186/s12906-020-02887-7

**Published:** 2020-03-19

**Authors:** Hassan Mahmoudi, Leili Shokoohizadeh, Nayreh Zare Fahim, Ali Mohamadi Bardebari, Shirin Moradkhani, Mohammad Yousef Alikhani

**Affiliations:** 1 Department of Microbiology, Faculty of Medicine, Medical Microbiology, Hamadan University of Medical Sciences, Hamadan, Iran; 2 School of Pharmacy, Hamadan University of Medical Sciences, Hamadan, Iran; 3 Brucellosis Research Center, Hamadan University of Medical Sciences, Hamadan, Iran

**Keywords:** *Acinetobacter baumannii*, Efflux pump, Menthol, *Mentha longifolia*

## Abstract

**Background:**

*Acinetobacter baumannii* is an opportunistic pathogen that causes nosocomial infections especially in patients in intensive care units (ICUs). Accordingly, the aim of our study was to detection *of adeABC* efllux pump encoding genes and antimicrobial effect of the essential oil of *Mentha longifolia* and *Menthol* on the minimum inhibitory concentration (MIC) of imipenem and ciprofloxacin in clinical isolates of *A. baumannii.*

**Methods:**

A total of 75 clinical isolates of *A. baumannii* were collected. The presence of efflux pump genes was detected by polymerase chain reaction (PCR). The minimum inhibitory concentration (MIC) of the essential oil of *Mentha longifolia* and Menthol and their combined effect with antibiotics were measured by microbroth dilution method and fractional inhibitory concentration (FIC) index.

**Results:**

The frequency of *adeA*, *adeB*, and *adeC* genes in clinical isolates of *A. baumannii* were 86.7, 90.7, and 92%, respectively. When the essential oil of *Mentha longifolia* was combined with ciprofloxacin and imipenem, MICs decreased 4- and 8-fold, respectively. In the combination of menthol with imipenem, the resistance to imipenem was reduced from 0- to 16-fold in 90% (63/70) of the isolates.

**Conclusion:**

The presence of efflux pump genes in more than 90% of *A. baumannii* isolates indicates its potential role in inducing imipenem- and ciprofloxacin-resistance in this bacterium. Menthol has an antimicrobial effect as an active ingredient in *Mentha longifolia*. In the future, the combination of medicinal plants with antibiotics can be used as a complement in treating diseases caused by drug-resistant bacteria such as *A. baumannii* infections.

## Background

*Acinetobacter baumannii* is known as one of the most important hospital pathogens. Controlling the infections caused by this bacterium has created many problems due to its multiple drug resistance. Therefore, antibiotic treatment has become a challenge for hospitalized patients, especially in intensive care units (ICUs) [1]. Efflux pumps are considered as one of the most important mechanisms of intrinsic and acquired antibiotic resistance in bacteria that can remove toxic substances such as antibiotics, drugs, and chemicals, as well as secretion of cellular products out of the cell. Efflux pumps prevent proper concentrations of toxic substances used to inhibit bacteria and, as a defence *mechanism* against harmful substances in the environment, allow bacteria to survive in different environments. Different antibiotics serve as substrates for different pumps. Efflux pumps are one of the intrinsic and acquired resistance pathways in bacteria that can cause resistance to a wide range of antibiotics and disinfectants. Mutations in the efflux genes are associated with their expression and consequently with increased antibiotic resistance [2, 3]. The AdeABC pump is one of the most important systems of the efflux family which belongs to the RND family and *adeA, adeB,* and *adeC* genes encode it. AdeA is a membrane fusion protein, AdeB is a multi-drug transporter, and AdeC is an outer membrane protein [1]. Many chemicals can enhance the expression of *adeABC* genes and results in resistance to aminoglycoside, fluoroquinolones, beta-lactams, chloramphenicol, and tetracycline antibiotics. Various inhibitors have been used to deactivate efflux pumps including *Phenylalanine*-*Arginine Beta*-*Naphthylamide* (PAβN), Naphthyl-methylpiperazine, and Carbonyl-Chlorophenyl Hydrazou which by affecting and controlling the AdeABC pump can prevent antibiotics from being expelled from bacteria. Thus, antibiotics reach the minimum inhibitory concentration (MIC) [4]. Because of the toxicity of most of these compounds in humans, their widespread use is prohibited. Hence, the new challenge is to find nontoxic compounds or compounds with less toxicity [4]. As a result, the elimination of chemical inhibitors and the use of natural inhibitors such as plant compounds have recently attracted much attention. One of the medicinal plants that have shown antimicrobial properties is *Mentha longifolia*. *Mentha longifolia* is a member of the *Laminacea* family which is a perennial herb. It consists of more than 25 species and grows wildly in humid regions of central and southern Europe, Southwestern Asia, the Mediterranean, and North Africa. The essential oil of the *Mentha longifolia* species has shown an antimicrobial activity (comparable to available antibiotics) against a range of microorganisms including bacteria, fungi, and protozoa [5]. Essential oils and their constituents have significant hydrophobic properties which cause materials to penetrate into the cell membranes of bacteria and mitochondria disrupting their structure and creating more permeability. This causes the leakage of ions and other contents from the cell [6]. So far, there has been no study about the inhibitory effects of *Mentha longifolia* on efflux pumps in *A. baumannii*. Due to the frequency and availability of this plant as well as its therapeutic usages, the aim of this study was to investigate the effect of natural herbal inhibitors such as *Mentha longifolia* on MICs of ciprofloxacin and imipenem in clinical isolates of *A. baumannii* with *adeABC* efflux pumps encoding genes isolated from hospitalized patients in ICU wards.

## Methods

### Bacterial strain

A total of 75 clinical isolates of *A. baumannii* were isolated from clinical samples of patients hospitalized in ICUs from January to August 2018. Seventy imipenem- and ciprofloxacin-resistant isolates were selected for the study.

### Antimicrobial susceptibility

The antibiotic susceptibility of *A. baumannii* to ciprofloxacin (5 μg) and imipenem (10 μg) was detected by disk diffusion and microbroth dilution methods according to clinical & laboratory standards institute (CLSI) criteria [7, 8]. *Pseudomonas aeruginosa* ATCC 27853 and *Escherichia coli* ATCC 25922 were used as control strains for antibiotic susceptibility testing.

### Detection of *adeABC* genes

The bacterial genomic DNAs were extracted from overnight cultures of *A. baumannii* isolates using a commercial DNA purification kit (Sinaclon Co, Tehran, Iran) according to the manufacturer’s protocol. All isolates were screened for the presence of efflux pump-encoded genes including *adeA*, *adeB*, *adeC* using a multiplex PCR technique. The sequences of primers [9] used in the present study have been shown in Table 1.

**Table 1:** The primers used in this study for detection of *adeABC* genes

Gene	Primer sequence (5′ 3′)		Amplicon size(bp)	Reference
*ade A*	**F**	ATCTTCCTGCACGTGTACAT	**513**	9
	**R**	GGCGTTCATACTCACTAACC		
*ade B*	**F**	TTAACGATAGCGTTGTAACC	**541**	9
	**R**	TGAGCAGACAATGGAATAGT		
*ade C*	**F**	TACGGACTGCTACGCTTAAT	**527**	9
	**R**	AACAGGATGACCTGCTAACA		

The PCR mix was prepared in the final volume of 25 μl containing 1 μl (10 pmol) of each primer, 2 μl template DNA, 12.5 μl PCR Master Mix, and distilled water. DNA amplification was conducted in a thermal cycler (S1000™, Bio-Rad, Hercules, CA, USA), under the following conditions: initial denaturation at 94 °C for 5 min, followed by 30 cycles of denaturation at 94 °C for 30 s, annealing temperature of 55 °C for 1 min, extension at 72 °C for 1 min, followed by a final extension at 72 °C for 6 min. The electrophoresis of the amplified DNA fragments, along with a 100 bp DNA ladder, was performed using agarose gel 1.5%. The size of the amplification fragment for *adeA*, *adeB* and *adeC* genes were 513, 541 and 527 bp, respectively.

### Preparation of the essential oil of *Mentha longifolia*

For this experimental study, the medicinal plant *Mentha longifolia* was collected from Alvand Mountains of Hamadan in the west of Iran, was identified in the School of Pharmacy of Hamadan University of Medical Sciences, and was *assigned* a *herbarium* code (herbarium code 37). The essential oil was obtained by hydro-distillation of air-dried leaves using a Clevenger-type apparatus for 3 h. Finally, the essential oil was obtained as a light yellow liquid. The obtained essential oil was dried over anhydrous sodium sulfate and, after filtration, stored in dark vials at 4 °C.

### Preparation of menthol solution

Menthol (ALDRICH, Lot BCBQ32755V) was purchased as powder from Sigma Aldrich Company. One μg of menthol was dissolved in 1 ml distilled water (as the solvent).

### Antibacterial activities of menthol and essential oil of *Mentha longifolia*

The essential oil of *Mentha longifolia* and Menthol with the final concentrations of 512 μg/ml were loaded on blank disks and their antibacterial activities against the clinical isolates of *A. baumannii* were investigated by the disk diffusion method*.* The diameter of blank disk was 6 mm and 50 μL from each dilution of *M. longifolia* essential oil and Menthol were added to the blank disks. The bacterial suspension with a turbidity equivalent to 0.5 McFarland (1.5 × 10^8^ CFU/ml) in the Brain Heart Infusion broth (BHI) (Merck, Germany) was prepared and cultured on a Muller-Hinton Agar (Merck, Germany) plate. The disk containing dimethyl sulfoxide (DMSO) was used as a solvent of essential oil and as the negative control. The plates were incubated at 37 °C for 24 h [10]. Finally, the antibacterial activity of the essential oil was evaluated by observing the inhibitory zones around the disks. The MICs of the essential oil *Mentha longifolia,* Menthol, imipenem and ciprofloxacin were determined by the broth microdilution method. According to CLSI guidelines the MICs of resistance to imipenem and ciprofloxacin in *Acinetobacter* isolates is ≥16 μg/ml and ≥ 4 μg/ml, respectively [8]. The tests were repeated three times to achieve the required accuracy.

### Assessment of the synergistic effects

The synergistic effects of *Mentha longifolia* essential oil and *Menthol*, imipenem, and ciprofloxacin were assessed using the microbroth dilution method. Serial dilutions from 512 to 0.25 μg/ml of each concentration were prepared in microtiter plates at the volume of 10 μl. Then, 90 μl of the bacterial suspension in the Brain Heart Infusion broth (BHI) media (Merck, Germany) was added to the microtiter plate to produce a final inoculum of 5 × 10^5^ CFU/ml. The plates were incubated for 24 h at 37 °C. For combining two antimicrobial agents, fractional inhibitory concentration index (FICi) was calculated as follows: [(MIC of drug A in combination) / MIC of drug A alone)] + [(MIC of drug B in combination) / (MIC of drug B alone)]. The FICi index was interpreted as follows: Synergy ≤0.5, Addition > 0.5 to 1.0, Indifference 1.0 *<* to *<* 4.0, and Antagonism ≥4.0 [11].

### Statistical analysis

This investigation was a descriptive-application study. SPSS software V 21 was used for the statistical analyses. The *P* value and confidence intervals were less than 0.05 and 95%, respectively.

## Results

### Antimicrobial susceptibility testing

**Fig. 1:**
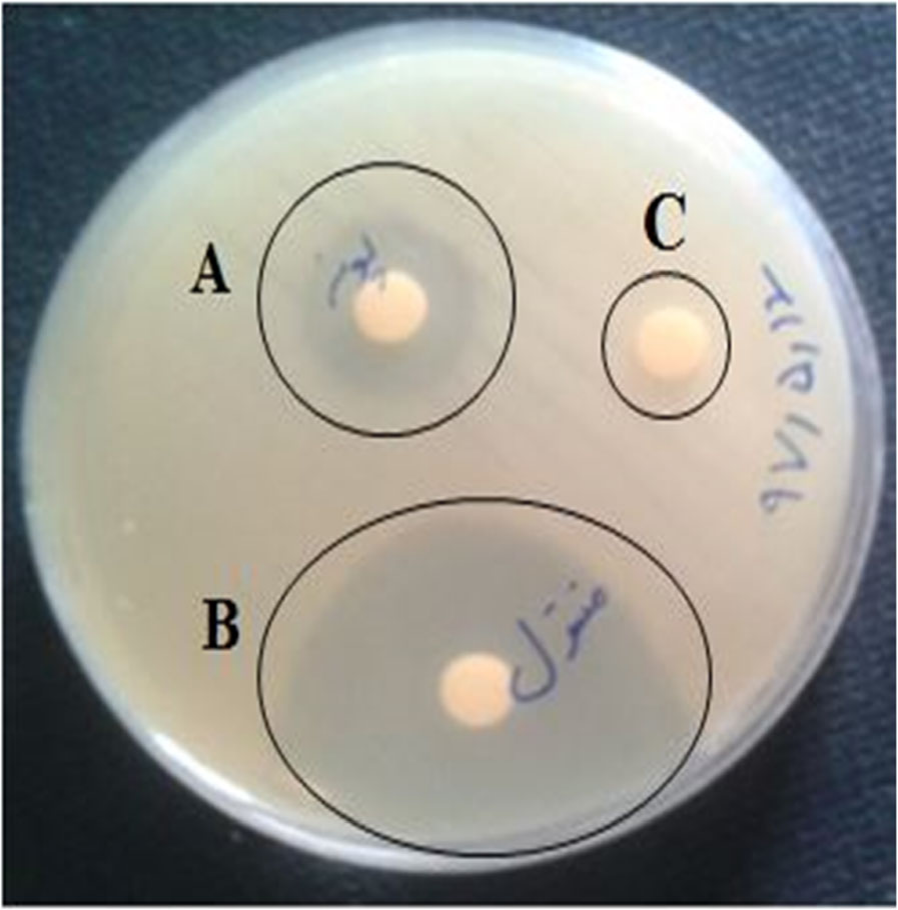
Inhibitory effect of *Mentha longifolia* essential oil and *Menthol* with the final concentrations of 512 μg/ml on *A. baumannii* isolate. **a**: *Mentha longifolia;***b**: *Menthol;***c**: Negative Control (DMSO)

The antibiogram results showed that 72 (97%) and 70 (94%) isolates of *A. baumannii* were resistant to ciprofloxacin and imipenem, respectively. According to the results of disk diffusion method, the antibacterial effects of menthol and the essential oil of *Mentha longifolia* were observed in the inhibition zones around disks (Fig. 1).

### Frequency of *adeABC* efflux pump genes

**Fig. 2:**
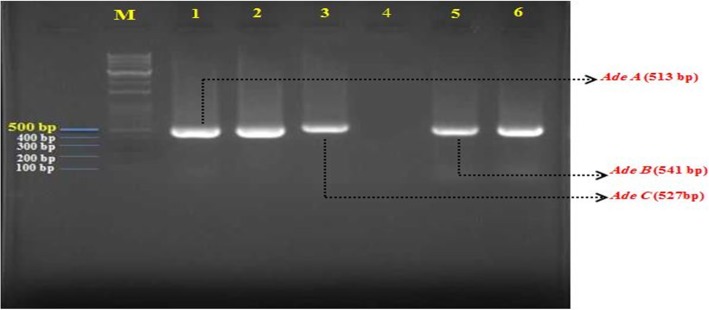
Gel electrophoresis of the efflux pupm *adeABC* genes of *A. baumannii* isolates. M: 100 bp DNA marker; Lane 1–2: *adeA*; Lane 3: *adeB*; Lane 4: Negative control. Lane 5–6: *adeC*

The multiplex PCR was successfully performed and 513 bp, 541 bp, and 527 bp bands were amplified as *adeA, adeB*, and *adeC* genes, respectively. The results showed that the frequencies of *adeA*, *adeB*, and *adeC* genes were 65(86.7%), 68(90.7%), and 69(92%), respectively (Fig. 2). The combination of these genes among the *A. baumannii* isolates included as *adeA* + *adeB* 62(82.7%), *adeA* + *adeC* 64(85.3%), *adeB* + *adeC* 67(89.3%), and *adeA* + *adeB* + *adeC* 62(82.7%). These results showed that the drug efflux systems are associated with resistance to ciprofloxacin and imipenem in clinical isolates of *A. baumannii*. These results showed that the drug efflux systems are associated with resistance to ciprofloxacin and imipenem in clinical isolates of *A. baumannii*.

### MIC and the synergistic effects of imipenem and ciprofloxacin in combination with *Mentha longifolia* and *Menthol*

The result showed that the MICs of imipenem and ciprofloxacin against clinical isolates of *A. baumannii* were between 8 and 128 μg/ml and 4–32 μg/ml, respectively (Tables 2, 3). The checkerboard method was used to assess synergism by calculating the FICi, which is an interaction coefficient that indicates whether the combined inhibitory/bacteriostatic effects of drugs are synergistic, additive, or indifferent. The means of FICi for all isolates in relation to *Mentha longifolia*-ciprofloxacin, *Menthol*–ciprofloxacin, *Mentha longifolia*-imipenem, and Menthol-imipenem were calculated as 3.5, 1.23, 0.40, and 1.23, respectively (Tables 2, 3). The FICi results suggest that the combined effect of imipenem-menthol and imipenem-*Mentha longifolia* against *A. baumannii* isolates is synergistic. The effects of the efflux pump inhibitors were determined by detecting a 4-fold or greater increase in susceptibility (reduction in the MICs) after incorporation of *Mentha longifolia* and *Menthol.* Totally, in the combination of antibiotics with *Mentha longifolia* and *Menthol*, the MICs for 25 out of 70 isolates (35.71%) were decreased significantly 4- to 32-fold. When the essential oil of *Mentha longifolia* was combined with ciprofloxacin and imipenem, MICs decreased 4- and 8-fold, respectively. In the combination of Menthol with imipenem, in 90% (63/70) of the isolates the resistance to imipenem was reduced from 0- to 16-fold.

**Table 2:** MICs and FICi of imipenem, *Mentha longifolia* and Menthol against *A. baumannii*

***A. baumannii*** isolates No (%)	MIC IMP (μg/ml)	MIC ***M. longifolia*** (μg/ml)	MIC (IMP+ ***M. longifolia)*** (μg/ml)	FICi	MIC Menthol (μg/ml)	MIC (IMP+ Menthol) (μg/ml)	FICi
**5 (7.15)**	8	2.5	0.5	0.26	4	0.25	0.09
**2 (2.85)**	16	2.5	1	0.46	4	0.5	0.1
**35 (50)**	32	2.5	2	0.86	4	1	0.28
**12 (17.15)**	64	2.5	4	1.66	4	2	0.5
**16 (22.85)**	128	2.5	8	3.26	4	4	1.03

The MICs of imipenem and ciprofloxacin in resistant *A. baumannii* was ≥16 (μg/ml) and ≥ 4 (μg/ml), respectively

**Table 3:** MICs and FICi of ciprofloxacin, *M. longifolia* and *Menthol* against *A. baumannii*

***A. baumannii*** isolates No (%)	MIC CIP (μg/ml)	MIC ***M. longifolia*** (μg/ml)	MIC (CIP + ***M. longifolia)*** (μg/ml)	FICi	MIC ***Menthol*** (μg/ml)	MIC (CIP + ***Menthol)*** (μg/ml)	FICi
**10 (14.2)**	4	2.5	2	1.3	4	1	0.5
**17 (24.6)**	8	2.5	4	2.1	4	2	0.75
**31 (50)**	16	2.5	8	3.7	4	4	1.25
**12 (17.4)**	32	2.5	16	6.9	4	8	2.25

## Discussion

In recent years, *A. baumannii* strains with multiple drug resistance patterns (MDR), extensive drug resistance (XDR), and pan drug resistance (PDR) have been increasing causing many problems for the treatment of patients infected with *A. baumannii* isolates [12, 13]. According to the results of some researches from Iran and other countries, 31% of bacterial isolates from patients admitted to ICUs were identified as *A. baumannii* [14, 15]. Based on the results of our research, more than 90% of *A. baumannii* isolates showed resistance to ciprofloxacin and imipenem. A study has shown that the resistance of *A. baumannii* not only to beta-lactams and carbapenems but also to other families of antibiotics including aminoglycosides and fluoroquinolones has been increasing [16]. Ardebili et al. reported that more than 100% of the *A. baumannii* isolates were resistant to ciprofloxacin, with MICs ranging from 4 to ≥128 μg/mL, however, the MIC values in the current study ranged from 4 to 32 μg/ml [17]. Resistance to ciprofloxacin is increasing in Iran and worldwide [18–20]. Nowak et al. showed that 97% of *A. baumannii* isolates which were previously cultured from respiratory tract samples from 15 hospitals in Greece, Italy, and Spain were resistant to imipenem [21]. Blitchtein et al. have also shown that 97.5% *of A. baumannii* isolated from a hospital in Lima, Peru were carbapenem-resistant [22]. The resistance of clinical isolates of *A. baumannii* to imipenem is rapidly increasing in Iran. A meta-analysis in Iran revealed that 55% of *A. baumannii isolates* were resistant to imipenem and 74% showed MDR phenotypes [23]. The results of studies in our area indicate that resistance to imipenem and other antibiotics is increasing over the time. In 2008, this rate was 16.3% but has reached 95% in the current study indicating that carbapenems are not a suitable choice for the treatment of *A. baumannii* infections [24]. Our findings highlight the critical need for a comprehensive monitoring and infection control policy as well as a national susceptibility review program that evaluates MDR *A. baumannii* isolates from different parts of Iran.

The results of our study were consistent with those of previous studies demonstrating the important role of efflux pumps in the resistance to ciprofloxacin. In our study, the frequencies of *adeA*, *adeB*, and *adeC* genes in clinical isolates of *A. baumannii* were 86.7, 90.7, and 92%, respectively. In accordance with our results, Japooni et al. detected *ade*A, *adeB*, and *adeC* genes in 100, 100, and 96.5% of *A. baumannii* isolates, respectively. In the study of Khayat et al., *adeA*, *adeB*, and *adeC* genes were detected in 100% of *A. baumannii* strains in Iran [18, 19]. Similar studies have also reported that the prevalence of these genes is from 53 to 97% [25–27]. In this study, the high frequency of *adeABC* genes in the *A. baumannii* isolates suggests that one of the mechanisms involved in creating a high resistance to ciprofloxacin and imipenem can be AdeABC efflux pumps. However, the expressions of genes coding for AdeABC efflux pumps were not assessed by the real-time PCR technique which is a limitation of the current study. It is relatively difficult both to determine if one or more efflux pumps have been inhibited and to identify the target efflux pump. A bacterium may possess many efflux pumps including several uncharacterized or even unidentified efflux pumps.

One of the main goals of this study was to investigate the effect of *Mentha longifolia* essential oil and menthol as natural efflux pump inhibitors compared with synthetic substances. Thus, using the essential oil of *Mentha longifolia* and menthol decreases the toxic risks of antibiotics [28]. Studies have shown that the essential oil of *Mentha longifolia* has antibacterial properties against Gram-positive and Gram-negative bacteria such as *Listeria monocytogenes, Staphylococcus aureus, Escherichia coli*, *Shigella spp.*, *Salmonella typhimurium*, and *Pseudomonas aerosinosa*. It is also known that the essential oil of *Mentha longifolia* has a higher antimicrobial effect than its alcoholic extract due to the presence of menthol, menthone, pepogon, isomenthol. Menthol is the most important compound of *Mentha longifolia* with its antimicrobial effects reported in various studies [29–32]. Combination therapy increases antimicrobial activity, minimizes antibiotic toxicity, prevents the occurrence of mutations involved in bacterial resistance during treatment, and also leads to a synergistic antimicrobial activity. In this study, the effect of synergism in the combination of menthol-imipenem and essential oil of *Mentha longifolia* with imipenem was greater than that of menthol-ciprofloxacin and essential oil *of Mentha longifolia* with ciprofloxacin. In addition, Menthol-imipenem significantly reduced the MIC of imipenem (16-fold). Moreover, *Mentha longifolia*-ciprofloxacin and *Mentha longifolia* decreased the MICs of ciprofloxacin and imipenem 4- and 8-fold, respectively. Guclo et al. studied the effect of menthol on MDR *A. baumannii* and found that menthol has an antimicrobial effect [29]. Seasotiya et al. investigated the inhibitory effect of 35 different herbal extracts on efflux pumps and showed that these extracts increased the accumulation of drugs in bacteria and reduced the efflux of fluoroquinolones [33].

In our study, menthol and essential oil of *Mentha longifolia* reduced the MICs of imipenem and ciprofloxacin 4- to 8-fold, which are lower than the results of synthetic efflux pump inhibitors such as Carbonyl Cyanide 3-Chlorophenylhydrazone (CCCP). Ardebili et al. showed that the susceptibility of *A. baumannii* strains to ciprofloxacin highly increased in the presence of efflux pump inhibitors (EPI) and CCCP reduced the MICs 2- to 64-fold [17]. Many compounds extracted from the plant were introduced as EPI which inhibited bacterial pathogens. These compounds affected different efflux pumps including: *MexAB-OprM* in *Pseudomonas aeruginosa*; *Nor A* in *Staphylococcus aureus*, *Bacillus cereus*, *Staphylococcus epidermidis,* and *Salmonella enteritidis*; ND in *Escherichia coli* and Food-borne pathogens; and *Msr A* and *Tet K* in *S. epidermidis* [34–42].

## Conclusions

According to our results, the essential oil of *Mentha longifolia* and menthol as its main constituent can be good candidates to investigate antimicrobial activities and potential efflux pump inhibitors in *A. baumannii*. Thus, natural and synthetic derivatives of medicinal plants can be considered as potential adjuvants to maintain the efficacy of antibiotics in the treatment of infectious diseases. However, more research is needed regarding the molecular interactions between these compounds and potential efflux pumps.

## Data Availability

The datasets used and analyzed in the current study are available from the corresponding author on reasonable request.

## Abbreviations

ICU: Intensive care units
PCR: Polymerase chain reaction
MIC: Minimum inhibitory concentration
FIC: Fractional inhibitory concentration
CLSI: Clinical & laboratory standards institute
BHI: Brain heart infusion broth
DMSO: Dimethyl sulfoxide
